# Congenital Epulis

**Published:** 2016-02-12

**Authors:** Joshua Halka, Kongkrit Chaiyasate

**Affiliations:** Beaumont Health System, Royal Oak, Michigan

**Keywords:** granular, cell, tumor, congenital, epulis

**Figure:**
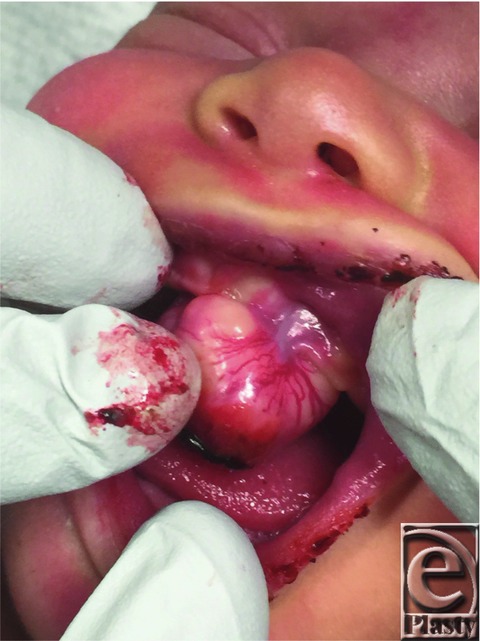


## DESCRIPTION

A female neonate was noted to have a mass on the left maxillary gingiva. The infant was unable to breast-feed, but there was no concern for airway compromise, so she was placed under general anesthesia and the mass was excised and left to heal by secondary intention.

## QUESTIONS

1. **What is congenital epulis?**
2. **How does congenital epulis commonly present?**
3. **What is thought to be the cause of congenital epulis and why?**
4. **What are the management options for congenital epulis?**

## DISCUSSION

Congenital epulis was first described by Neumann in 1871 and as a result has been called Neumann's tumor.^1^^-^8 The Greek term “epulis” means “on the gingiva.” It is more specifically called “congenital granular cell epilus,” and this is the title recommended by the World Health Organization.^5^ It has also been called congenital granular cell lesion, gingival granular cell tumor of the newborn, congenital epilus of the newborn, congenital granular cell myoblastoma, and granular cell fibroblastoma. It has been estimated that the incidence of congenital epulis is 0.0006%, with multiple medical centers reporting only a few cases or less over multiple decades.^4^

Congenital epulis commonly presents in neonates, with a mass noted to arise from the gingiva. The lesions are often covered with smooth pink to red mucosa and are sometimes ulcerated. The most common location is the anterior part of the maxillary alveolar ridge, usually in the region of the lateral incisors or canines. It has been reported to arise from the mandibular gingiva or tongue, as well as at multiple locations simultaneously.^2^^,^^4^^,^^7^ It is estimated that it is 2 to 3 times more common to occur on the maxilla than on the mandible, and 10% of the time multiple lesions may occur simultaneously.^1^^,^^2^^,^^4^^,^^5^^,^^7^ Congenital epulis more commonly occurs in female newborns than in male newborns, with an estimated ratio of 9-10:1 female to male predominance. Congenital epulis is typically a few millimeters to a few centimeters in size but has been described to be as large as 9 cm.^1^^-^7

The cause of congenital epulis is unknown. Microscopically, it is often composed of large sheets or ribbons of polygonal or rounded cells with a small, dark basophilic nucleus and eosinophilic granular cytoplasm. There is often a prominent vascular network and overlying stratified squamous epithelium with the absence of rete ridges. Immunohistochemical studies show positivity for vimentin but will be negative for S-100 and laminin. Adult granular cell tumors are considered to arise from Schwann cells and stain positive for S-100 and laminin. There are multiple theories as to the pathogenesis of congenital epulis. One theory is that given the female predominance, the growth of tumor stopping after birth, and the fact that some tumors have been shown to spontaneously regress, the cause may be influenced by maternal or fetal hormones during pregnancy; however, estrogen and progesterone receptors have not been found to be positive in congenital epulis.^2^^-^5^,^^8^ Ultrastructural and immunohistochemical findings in reported cases have supported a mesenchymal origin of congenital epulis, as the cells have features of histiocytes and fibroblasts.^1^^-^3^,^^6^

Congenital epulis has classically been managed by complete surgical excision under general or local anesthesia. There has also been a case report of CO^2^ laser use with general anesthesia. Congenital epulis has never been reported to undergo malignant transformation or to continue growth after birth. Even with incomplete excision, no recurrence has been reported. Spontaneous regression has been reported when surgical management is not attempted.^1^^-^6^,^^8^ Congenital epulis has been diagnosed by prenatal ultrasonography but has never been reported to be diagnosed by ultrasonography prior to 26 weeks of gestation. On the basis of this, it has been theorized that it starts growing after this point in pregnancy. Magnetic resonance imaging can be used for diagnosis and surgical planning in congenital epulis suspected during pregnancy as it is more accurate than ultrasound. If congenital epulis is not interfering with feeding or respiration, nonsurgical management can be considered. But if these issues arise, surgical intervention should be strongly considered.^1^^-^2^,^^8^

## SUMMARY

Congenital Epilus is a rare, benign gingival mass that occurs in newborns. It can be managed non-operatively or via excision. If the mass is causing issues with feeding or respiration, intervention is recommended.
